# Sun-Tanning Perceptions of a New Zealand Urban Population (1994–2005/6)

**DOI:** 10.1155/2014/135473

**Published:** 2014-02-10

**Authors:** A. I. Reeder, G. F. H. McLeod, A. R. Gray, R. McGee

**Affiliations:** ^1^Cancer Society of New Zealand Social & Behavioural Research Unit, Department of Preventive & Social Medicine, Dunedin School of Medicine, University of Otago, P.O. Box 913, Dunedin 9054, New Zealand; ^2^Department of Psychological Medicine, School of Medical and Health Sciences, University of Otago, Christchurch 8140, New Zealand; ^3^Department of Preventive & Social Medicine, Dunedin School of Medicine, University of Otago, P.O. Box 913, Dunedin 9054, New Zealand

## Abstract

*Background*. Sun-tanning perceptions are monitored to identify changes and help refine targeting of skin cancer prevention messages. *Aim*. To investigate associations between perceptions of sun-tanning and demographic factors among a New Zealand urban population, 1994–2006. *Methods*. A telephone survey series was conducted during summer in 1994, 1997, 1999/2000, 2002/2003, and 2005/2006. Demographic and personal information (sex, age group, skin sun-sensitivity, and self-defined ethnicity) obtained from 6,195 respondents, 50.2% female, 15–69 years, was investigated in relation to six sun-tanning related statements. A total “positive perceptions of tanning” (ProTan) score was also calculated. Regression analyses modelled each component and the ProTan score against survey year and respondent characteristics. *Results*. Statistically significantly higher ProTan scores were found for age group (strong reverse dose-response effect), male sex, residence (highest in Auckland), ethnicity (highest among Europeans), and sun sensitivity (an *n*-shaped association). There was no statistically significant change in total ProTan scores from baseline. *Conclusions*. The development, pretesting, and evaluation of messages for those groups most likely to endorse ProTan statements should be considered for the New Zealand skin cancer prevention program. To achieve and embed significant change, mass media campaigns may require greater intensity and reinforcement with sustained contextual support for settings-based behavioural change.

## 1. Introduction

In environments where high levels of ambient solar ultraviolet radiation (UVR) are recorded, up to 95% of cutaneous malignant melanoma (melanoma) and 99% of other skin cancers are attributed to excess sun exposure [1]. New Zealand (NZ) has rates among the highest age-standardized incidence and mortality rates for cutaneous malignant melanoma [2], and recent registration rates show an upward trend, 1999–2010 [3]. In 2010, melanoma was the fourth most commonly registered cancer and resulted in 324 deaths among a total population of around 4 million. The most recent official estimate of public melanoma treatment costs is NZ$24.4 M/year [4]. Although the registration of nonmelanoma skin cancers (NMSC) is not required in NZ, there are an estimated 67,000 new cases per year, for which annual health system treatment costs are conservatively estimated to exceed NZ$48 M/year [5]. In addition, there is the cost of treating other solar UVR related diseases, such as cortical cataracts [6]. Although some UVR exposure is required to protect against bone diseases, such as rickets, osteomalacia, and osteoporosis, it has been argued that “there should be no need to accept an increased risk of diseases of excessive exposure, in order to achieve minimal risk of diseases of underexposure” [7].

Perceptions that a suntan is attractive and healthy may reinforce sunbathing and contribute to excessive sun exposure [8]. Perceptions regarding the attractiveness of a tan are strongly correlated with sunbathing [9, 10]. Given the potentially modifiable nature of such perceptions, their conversion into sun protective attitudes among populations at-risk of skin cancer may play an important role in behavioural changes that would help reduce skin cancer risk [11].

Public health campaigns aimed at reducing excessive UVR exposure and increasing the frequency of sun protective behaviours were first developed in Australia. The original campaign slogan, “Slip (on a shirt), Slop (on sunscreen), Slap (on a hat)” was launched in 1981 [12]. Although protanning attitudes continued to be commonly held, especially among males and younger respondents [13], subsequent Victorian survey research concluded that the campaign appeared to be effective, with positive perceptions of tanning decreasing significantly from 1988 to 1990 [14]. By 1998, the percentage of respondents that liked to get a suntan had reduced from 61% in 1988 to 35% [15]. Campaigns using mass media which were initiated in other countries produced inconclusive results regarding attitude change, although there were some encouraging findings [16].

In NZ, national and regional health promotion programs aimed at increasing awareness of skin cancer and reducing excessive solar UVR exposure were implemented in 1988 [17]. Since it was important to evaluate these efforts, the Cancer Society of New Zealand Inc. (CSNZ) and the Health Sponsorship Council (now the Health Promotion Agency, HPA), initiated the Triennial Sun Protection Survey (Sun Survey) series, modelled on Victorian precedent [13], with data collected about the sun protection knowledge, perceptions, and practices of the NZ urban population. Selected findings published from the first two surveys, 1994 and 1997, indicated that appropriate use of sun protection was poor, resulting in high levels of sunburn [18], in particular, among younger age groups [19, 20]. Thereafter, the overall frequency of self-reported, summer weekend sunburn continued to exceed 20% [21].

The five waves of data in this unique Sun Survey database also provide opportunities to investigate perceptions regarding tanning. The aims of the present study were to investigate among the NZ urban population, 1994–2006, (1) six specific dimensions of sun-tanning perceptions, (2) a summed ProTan score, and (3) associations between these measures and respondent characteristics (city of residence, sex, age group, skin sun-sensitivity, and self-reported ethnicity) and survey year. It was hypothesized that population perceptions might change over time and differ by these demographic characteristics, with some groups having more positive perceptions than others and thereby increasing their potential future risk of skin cancer. Insights obtained from the study could potentially inform and help guide the existing SunSmart program and the content and targeting of future skin cancer prevention efforts.

## 2. Methods

### 2.1. Sample Selection

Respondents, aged 15–69 years inclusive, were resident in households randomly selected (using random digit dialling in predetermined areas, 1994 and 1997, or telematched from electoral rolls, 1999–2006) in five metropolitan areas: Auckland, Wellington, Hamilton, Christchurch, and Dunedin, which represented approximately 55% of the total NZ resident population in the 2006 Census. The random selection procedure was limited to respondents from around 92% of NZ households with land-line telephone access around this time [22]. Given a primary prevention focus, interview protocols prioritised younger household members, but a quota system ensured that the sample comprised approximately equal numbers of each sex, and that each city contributed 20%, both of adolescents (15–17 years) and adults (18–69 years).

### 2.2. Procedures

Meteorological data were used to select appropriate survey weekends during southern hemisphere summers, with the main criterion being that the weather had been sufficiently “fine” for potentially harmful sun exposure to have occurred [18]. The telephone questionnaire was administered by market research contractors using computer assisted telephone interviewing (CATI) systems. Interviews were usually conducted on either a Monday or Tuesday evening, following the selected survey weekends.

### 2.3. Measures

Respondents were administered a questionnaire concerning weekend sun exposure and sun protective behaviours which also included demographic information and measures of sun-tanning perceptions. For the latter, respondents were asked to rate, on a five-point Likert-type scale, their level of agreement or disagreement (1 = Strongly disagree; 2 = Disagree; 3 = Neither agree or disagree; 4 = Agree; 5 = Strongly agree) with six statements: (1) “I feel more healthy with a suntan” (hereafter abbreviated to *More Healthy*); (2) “a suntan makes me feel better about myself” (*Feel Better*); (3) “a suntan makes me feel more attractive to others” (*More Attractive*); (4) “this summer I intend to sunbathe regularly to get a suntan” (*Intention*); (5) “most of my close family think that a suntan is a good thing” (*Family*); and (6) “most of my friends think a suntan is a good thing” (*Friends*). The content of these statements was guided by Australian research [13]. An investigation of the psychometric properties of the summative ProTan scale, constructed from these six items, supported its applicability to the NZ urban population [23]. A higher ProTan score indicates more positive perceptions of tanning.

Self-defined ethnicity was coded according to Level 1 (the highest) of the NZ Ministry of Health ethnicity and data protocols as either Māori, Pacific, Asian, or New Zealand European/European/Other (NZE/O) [24]. Self-reported skin type was based on a modified Fitzpatrick classification of skin sun-reaction: Type I (always burn, never tan), Type II (usually burn, tan with difficulty), Type III (sometimes burn, tan moderately), and Type IV (rarely burn, tan easily) [25].

### 2.4. Statistical Analyses

Responses to the six statements (*More Healthy*, *Feel Better*, *More Attractive*, *Intention*, *Friends*, and *Family*) were dichotomised into two categories, one of which included the Strongly disagree and Disagree responses, and the other which included the Strongly agree, Agree, and Neither Agree nor Disagree responses. Noncommittal respondents were included in the latter group because they did not express the preferred response, which was explicit disagreement with each ProTan statement. Responses to the six statements were modelled using logistic regression against survey year and respondent characteristics (city of residence, age, sex, self-defined ethnicity, and skin sun sensitivity). In addition, a total ProTan score was calculated by summing all six statement responses, creating a score between 6 and 30, and modelled using linear regression. All statistical analyses were performed using Stata 12.1 software and a two-sided *P* < 0.05 was considered statistically significant in all cases [26].

### 2.5. Ethical Approval

Participation in the survey was taken as informed consent. Participants had previously been notified of the survey by mail from the commissioned market research agency. The proposed project analyses, in part reported here, were reviewed and ethical approval granted at the Departmental level, following University of Otago Human Ethics Committee procedures.

## 3. Results

Data usable for analysis were obtained from 6,195 respondents (Table 1).

There were approximately equal numbers of participants by year, city of residence, and sex but relatively greater numbers of younger than older adults as a result of the primary prevention focus of study protocols. Overall, 80% of participants defined themselves as being either skin type I or II, the two groups most vulnerable to UVR skin damage. Respondents of non-European ethnicity were somewhat underrepresented in relation to the 2006 Census population.

The reference groups, odds ratios, and 95% confidence intervals for the responses to the six statements about sun-tanning perceptions by survey year and respondent characteristics are presented in Tables 2 and 3, both unadjusted and adjusted for all other tabulated variables. We now highlight key results, following the order of tabular presentation of the variables.

### 3.1. Survey Year and City of Residence

In the unadjusted model, survey year was positively associated with *Friends*, but this association was no longer significant after adjustment and survey year became statistically significantly associated only with the *More Attractive* variable, demonstrating a steadily strengthening positive relationship from 1999-2000 to 2005/6. Statistically significant differences between cities were found for* More Healthy*, *Feel Better* and *Friends*, with higher odds of endorsement of *More Healthy* and *Feel Better* by Auckland residents than those of other cities, with the *Feel Better* association weakening somewhat after adjustment. Christchurch residents were the least likely to endorse these statements. For *Friends*, all cities except Christchurch had higher odds of endorsement than Auckland.

### 3.2. Personal Characteristics

Compared with males, females had consistently significantly reduced odds of endorsing the *Healthy*, *More Attractive*, *Family*, and* Friends* statements, both before and after adjustment. With respect to age group, the odds of endorsing the *More Healthy*, *Feel Better*, *More Attractive*, *Friends*, and *Intend* statements demonstrated an almost entirely consistent reverse dose-response effect by decreasing significantly with increasing age, with only a few relatively minor exceptions in point estimate increments. For example, for *More Healthy*, the 20–29 year age group demonstrated slightly higher odds of endorsement than the youngest age group. Reporting the most sun-sensitive skin type was associated with the lowest odds of endorsing each statement, with the exception that the numerically small, least sensitive group had some lower odds, including lower adjusted odds for the adjusted *Friends* and *More Attractive* statements. Respondents of NZ European ethnicity had significantly higher odds of endorsing the* Feel Better* and *More Attractive* statements, for which those of Asian ethnicity had the lowest odds. The association between ethnicity and *Feel Healthy* only became statistically significant after adjustment, with Asians again having the lowest odds. The pattern for *Family* was less clear, but those of Māori ethnicity had somewhat higher odds of endorsement than Europeans, in both the unadjusted and adjusted models. Along with those of Pacific ethnicity, Māori had significantly higher odds, both unadjusted and adjusted, of endorsing the statement *Friends*, but the significantly increased unadjusted odds for *Intentions* were not found after adjustment. The odds of endorsing *Friends* were also somewhat higher among Asians than Europeans, also for *Intentions*, but in the latter case not after adjustment.

The associations between each of the six sample characteristics and the total mean ProTan score (range from 6 to 30) are presented in Table 4.

Before adjustment, all six sample characteristics were statistically significantly associated with ProTan score, but this association failed to reach significance for survey year after adjustment for the other five characteristics. In the adjusted model, mean ProTan scores peaked in 1999/2000 then declined, but there was no evidence of significantly less endorsement of tanning in 2005/6 than in 1994. Auckland residents had the highest and Christchurch residents the lowest mean ProTan score. Females had a significantly lower mean ProTan score than males, particularly after adjustment, and a strong reverse dose response effect was observed for age. As skin sun sensitivity reduced, ProTan scores increased, except among the relatively numerically small, least sun sensitive group. European ethnicity was the most strongly positively associated with ProTan score, whereas Asian ethnicity was the most strongly negatively associated.

## 4. Discussion

This is the first published study to report perceptions of sun tanning among the NZ urban population and investigate demographic and personal factors associated with them, based on all five surveys in the Sun Survey series, 1994–2006. Unlike what has been reported for Victoria, Australia [27], there was no evidence of statistically significant overall improvement in perceptions of tanning among the NZ population since baseline.

In multivariable analyses, city of residence, age group, sex, skin type, and ethnicity were each statistically significantly associated with mean ProTan score. Auckland residents were significantly more ProTan than other groups. Since Auckland is NZ's most populous city, is the most northern city surveyed, and has a tendency towards higher UVR levels than the other cities, there would seem to be a specific need for efforts to moderate ProTan perceptions there.

The strong, reverse dose response association between ProTan scores and age group is consistent with Victorian survey findings [27]. In Victoria, the response to this observed pattern was to initiate more “hard-hitting messages with shock value,” a mass media approach to targeting young adults which was backed up by local qualitative research. In New Zealand, at least during the survey series period, 1994–2006, the core mass media approach was to target caregivers and young children using animal exemplars and animated cartoons about sun protection. This content was likely to have had little appeal to the young adults most at risk and who, by design, were overrepresented in the survey series. Although some hard-hitting messages were used, these were the exception and mostly disseminated either prior to or early in the survey period.

NZ males expressed significantly more ProTan perceptions than females, both overall, and for four of the six measures, except *Intend* (intention to tan) and *Feel Better*. Respondents of NZ European ethnicity had a significantly higher mean overall ProTan score than all other ethnic groups, with those of Asian ethnicity having the lowest mean scores, consistent with negative social associations with skin darkening in, for example, Chinese culture [28]. Reports from Australia, the region with climatic and social conditions most readily comparable to NZ, have not reported analyses by ethnicity. In NZ, those of European ethnicity, especially males, are a key target group for changing positive perceptions of tanning, in particular, since they are likely to have the skin types most vulnerable to UVR damage.

Higher odds of endorsing positive statements about tanning would not necessarily be problematic, provided that the intention to sunbathe remained low. However, no significant change in intentions was observed since 1994. Sun bathing intentions are factors that will be important to continue to monitor in the Sun Exposure Survey (SES) which superseded the series reported here. In a descriptive report of the 2010 SES survey, around the same percentage of respondents endorsed the sunbathing intentions statement as in 1994 [29], although revised survey procedures limit the appropriateness of direct comparison.

Some limitations of this research need to be considered. First, it may only be appropriate to generalise our findings to the NZ urban population. Nevertheless, the five cities surveyed contributed more than 55% of the total population and, according to the 2006 Census, 73% of the resident population lived in the greater urban areas of NZ. Second, the present study and the Australian studies cited sometimes used slightly differently worded perception and demographic variables which may limit comparability. Third, although the NZ skin cancer awareness programme began in 1988, no baseline measures of perceptions were obtained until 1994, which leaves open the possibility that positive change may have occurred during the first six years of the programme, after which time it may have become more difficult to change the remaining, perhaps more entrenched, attitudes. This illustrates the need for adequate funding to support essential programme evaluation, including the taking of timely baseline measurements. Finally, it is possible that some questions may have been misinterpreted by some groups, perhaps due to language and cultural differences.

Further analyses are planned, in particular, regression models to identify which factors (in addition to personal characteristics and perceptions) may be most strongly associated with poor sun protection and sunburn experience [21], so that these factors may be targeted in prevention campaigns. These analyses will include climatic variables and contextual data, such as engagement in different types of activity.

## 5. Conclusions

Overall, NZ population ProTan perceptions in 2006 were not significantly different from those in 1994. Without sustained, significant, targeted public health investment in sun safety interventions, attitudinal and behavioural change is unlikely to occur. However, the guiding Australian SunSmart programme model has demonstrated a positive cost benefit ratio, such that “sustained modest investment in skin cancer control is likely to be an excellent value for money” [30].

Systematic reviews of skin cancer primary prevention interventions indicate that there is convincing evidence for the effectiveness of multicomponent, community-wide programmes with supportive media messages [31], but not mass media campaigns, alone [32]. Media campaigns focused on changing personal perceptions need reinforcement by building contextual support for attitudinal and behavioural change through changes in public policies and practices [33] and settings-based interventions, for example, in primary schools [34] and workplaces—contexts for which there is convincing evidence of effectiveness in improving sun protection behaviours [35, 36].

Finally, since perceptions differed significantly by respondent characteristics, NZ skin cancer prevention programs should consider development and evaluation of efforts specifically targeted towards those groups most likely to endorse and indicate intentions to partake in risky sun exposure behaviours or subscribe to perceived positive social norms for tanning.

## Figures and Tables

**Table 1:** Sample demographic and personal characteristics (*n* = 6,195).

Variable	*n*	%
Survey year		
1994	1,243	20.1
1997	1,188	19.2
1999/2000	1,250	20.2
2002/2003	1,250	20.2
2005/2006	1,264	20.4
City of residence		
North Island		
Auckland	1,254	20.2
Hamilton	1,237	20.0
Wellington	1,230	19.9
South Island		
Christchurch	1,242	20.1
Dunedin	1,232	19.9
Sex		
Male	3,084	49.8
Female	3,111	50.2
Age group (year range)		
15–19	756	12.2
20–29	1,270	20.5
30–39	1,416	22.9
40–49	1,109	17.9
50–59	999	16.1
60–69	645	10.4
Skin type*		
Most sun sensitive		
I	1,494	24.4
II	3,432	56.1
III	1,109	18.1
Least sun sensitive		
IV	84	1.4
Missing data	*76 *	
Self-defined ethnicity		
NZ European	5,326	86.7
M$\overset{-}{\text{a}}$ori	405	6.6
Pacific	123	2.0
Asian	231	3.8
All other	55	0.9
Missing data	*55 *	

*Modified Fitzpatrick sun-sensitivity scale.

Percentages may not total 100% due to rounding.

**Table 2:** Unadjusted and adjusted* odds ratios and 95% confidence intervals for personal perceptions by sample characteristics.

	More healthy				Feel better				More attractive			
	Unadjusted		Adjusted		Unadjusted		Adjusted		Unadjusted		Adjusted	
			*n* = 5,959				*n = *5,985				*n* = 5,913	
		*P* = 0.637		*P* = 0.524		*P* = 0.524		*P* = 0.633		*P* = 0.797		*P* = 0.023
Year (summer)												
1994	1.00		1.00		1.00		1.00		1.00		1.00	
1997	1.00	0.85, 1.17	1.03	0.87, 1.21	0.92	0.79, 1.08	0.95	0.80, 1.12	0.93	0.79, 1.10	0.98	0.83, 1.16
1999/2000	1.09	0.93, 1.28	1.23	1.04, 1.45	0.94	0.81, 1.11	1.07	0.91, 1.26	0.92	0.78, 1.08	1.11	0.93, 1.31
2002/2003	0.98	0.84, 1.15	1.08	0.91, 1.27	0.96	0.82, 1.13	1.06	0.90, 1.26	0.95	0.81, 1.11	1.13	0.95, 1.33
2005/2006	0.97	0.83, 1.14	1.11	0.94, 1.31	0.87	0.74, 1.02	1.02	0.87, 1.21	0.99	0.85, 1.16	1.28	1.08, 1.51

		*P* < 0.001		*P* < 0.001		*P* = 0.045		*P* = 0.016		*P* = 0.154		*P* = 0.066
City (N to S)												
Auckland	1.00		1.00		1.00		1.00		1.00		1.00	
Hamilton	0.73	0.62, 0.86	0.71	0.60, 0.84	0.88	0.75, 1.03	0.84	0.71, 0.99	0.92	0.79, 1.08	0.90	0.77, 1.07
Wellington	0.82	0.70, 0.96	0.79	0.67, 0.93	0.91	0.78, 1.07	0.88	0.74, 1.03	0.97	0.83, 1.14	0.95	0.80, 1.12
Christchurch	0.71	0.60, 0.83	0.68	0.58, 0.81	0.78	0.67, 0.92	0.75	0.64, 0.88	0.83	0.70, 0.97	0.79	0.67, 0.93
Dunedin	0.81	0.69, 0.94	0.78	0.66, 0.92	0.91	0.77, 1.06	0.87	0.74, 1.03	0.90	0.77, 1.05	0.87	0.74, 1.03

		*P* < 0.001		*P* = 0.009		*P* = 0.066		*P* = 0.149		*P* = 0.001		*P* = 0.009
Sex												
Male	1.00		1.00		1.00		1.00		1.00		1.00	
Female	0.84	0.76, 0.93	0.87	0.78, 0.97	0.91	0.82, 1.01	0.93	0.83, 1.03	0.85	0.77, 0.94	0.87	0.78, 0.97

		*P* < 0.001		*P* < 0.001		*P* < 0.001		*P* < 0.001		*P* < 0.001		*P* < 0.001
Age group												
15–19	1.00		1.00		1.00		1.00		1.00		1.00	
20–29	1.01	0.84, 1.21	1.04	0.86, 1.25	0.83	0.69, 1.00	0.83	0.69, 1.01	0.91	0.76, 1.09	0.91	0.76, 1.10
30–39	0.84	0.70, 1.01	0.86	0.72, 1.03	0.71	0.59, 0.85	0.68	0.56, 0.82	0.68	0.57, 0.81	0.63	0.53, 0.76
40–49	0.85	0.71, 1.03	0.87	0.72, 1.06	0.63	0.52, 0.76	0.59	0.49, 0.72	0.60	0.50, 0.73	0.54	0.45, 0.66
50–59	0.72	0.59, 0.87	0.72	0.59, 0.88	0.61	0.50, 0.74	0.59	0.48, 0.72	0.52	0.43, 0.63	0.47	0.38, 0.57
60–69	0.62	0.50, 0.76	0.63	0.50, 0.79	0.48	0.38, 0.59	0.44	0.35, 0.55	0.39	0.31, 0.49	0.35	0.27, 0.44

		*P* < 0.001		*P* < 0.001		*P* < 0.001		*P* < 0.001		*P* < 0.001		*P* < 0.001
Skin type												
I	1.00		1.00		1.00		1.00		1.00		1.00	
II	1.78	1.57, 2.02	1.75	1.54, 1.99	1.72	1.52, 1.95	1.71	1.51, 1.94	1.67	1.47, 1.89	1.64	1.44, 1.86
III	2.00	1.70, 2.34	2.03	1.72, 2.40	1.55	1.32, 1.81	1.66	1.41, 1.96	1.45	1.24, 1.70	1.53	1.29, 1.80
IV	1.00	0.62, 1.61	1.13	0.69, 1.84	0.96	0.61, 1.51	1.17	0.73, 1.87	0.74	0.45, 1.21	0.91	0.54, 1.51

		*P* = 0.825		*P* = 0.012		*P* < 0.001		*P* < 0.001		*P* < 0.001		*P* < 0.001
Ethnicity												
NZ European	1.00		1.00		1.00		1.00		1.00		1.00	
M$\overset{-}{\text{a}}$ori	0.98	0.80, 1.21	0.80	0.65, 0.99	0.82	0.67, 1.00	0.66	0.54, 0.82	0.82	0.67, 1.01	0.64	0.52, 0.79
Pacific	1.01	0.70, 1.47	0.72	0.49, 1.05	0.69	0.48, 0.98	0.48	0.33, 0.70	0.79	0.55, 1.14	0.53	0.36, 0.77
Asian	0.86	0.65, 1.13	0.67	0.50, 0.90	0.57	0.43, 0.75	0.45	0.34, 0.61	0.51	0.39, 0.69	0.37	0.27, 0.51
Other	0.85	0.49, 1.46	0.77	0.44, 1.34	0.66	0.38, 1.12	0.60	0.35, 1.04	0.50	0.28, 0.88	0.45	0.25, 0.80

*Adjusted for all six ProTan scale components, that is, all those listed in Tables 2 and 3, inclusive.

**Table 3:** Unadjusted and adjusted* odds ratios and 95% confidence intervals for perceptions of others and intentions statements.

	Family				Friends				Intentions			
	Unadjusted		Adjusted *n* = 5,816		Unadjusted		Adjusted *n* = 5,738		Unadjusted		Adjusted *n* = 6,021	
		*P* = 0.685		*P* = 0.212		*P* < 0.001		*P* = 0.397		*P* = 0.084		*P* = 0.314
Year (summer)												
1994	1.00		1.00		1.00		1.00		1.00		1.00	
1997	1.04	0.88, 1.23	1.06	0.89, 1.26	1.10	0.93, 1.30	1.13	0.95, 1.35	1.01	0.81, 1.25	1.04	0.83, 1.30
1999/2000	1.11	0.94, 1.31	1.19	1.00, 1.42	0.81	0.69, 0.96	0.99	0.83, 1.18	1.00	0.81, 1.24	1.26	1.01, 1.58
2002/2003	1.10	0.93, 1.29	1.20	1.00, 1.43	0.84	0.71, 0.99	1.12	0.94, 1.34	0.83	0.67, 1.04	1.07	0.85, 1.35
2005/2006	1.02	0.86, 1.20	1.13	0.95, 1.35	0.77	0.66, 0.91	1.07	0.89, 1.27	0.80	0.64, 1.00	1.10	0.87, 1.39

		*P* = 0.512		*P* = 0.815		*P* = 0.002		*P* = 0.004		*P* = 0.468		*P* = 0.588
City (N to S)												
Auckland	1.00		1.00		1.00		1.00		1.00			
Hamilton	0.95	0.81, 1.13	0.94	0.80, 1.12	1.12	0.95, 1.32	1.12	0.95, 1.34	0.82	0.66, 1.02	0.88	0.71, 1.11
Wellington	1.04	0.88, 1.22	1.00	0.85, 1.19	1.07	0.91, 1.26	1.07	0.90, 1.27	0.91	0.74, 1.13	0.94	0.75, 1.18
Christchurch	0.90	0.76, 1.06	0.92	0.77, 1.09	0.84	0.71, 0.99	0.89	0.75, 1.06	0.88	0.71, 1.09	0.84	0.67, 1.06
Dunedin	0.97	0.82, 1.14	0.97	0.81, 1.15	1.11	0.95, 1.31	1.23	1.04, 1.47	0.95	0.77, 1.17	0.98	0.78, 1.22

		*P* < 0.001		*P* < 0.001		*P* < 0.001		*P* < 0.001		*P* = 0.822		*P* = 0.165
Sex												
Male	1.00		1.00		1.00		1.00		1.00		1.00	
Female	0.70	0.63, 0.77	0.71	0.64, 0.80	0.77	0.70, 0.86	0.82	0.73, 0.92	1.02	0.88, 1.17	1.11	0.96, 1.28

		*P* < 0.001		*P* < 0.001		*P* < 0.001		*P* < 0.001		*P* < 0.001		*P* < 0.001
Age group (yrs)												
15–19	1.00	1.00	1.00		1.00		1.00		1.00	1.00	1.00	
20–29	0.56	0.47, 0.68	0.57	0.47, 0.69	0.45	0.36, 0.57	0.46	0.37, 0.58	0.53	0.43, 0.65	0.54	0.44, 0.67
30–39	0.41	0.34, 0.50	0.42	0.35, 0.50	0.24	0.20, 0.30	0.25	0.20, 0.32	0.31	0.25, 0.39	0.31	0.25, 0.39
40–49	0.47	0.39, 0.57	0.48	0.39, 0.58	0.21	0.16, 0.26	0.22	0.17, 0.27	0.25	0.19, 0.31	0.25	0.19, 0.32
50–59	0.46	0.38, 0.56	0.47	0.38, 0.57	0.16	0.12, 0.20	0.17	0.13, 0.21	0.25	0.19, 0.32	0.25	0.20, 0.33
60–69	0.43	0.35, 0.54	0.43	0.34, 0.54	0.13	0.10, 0.16	0.13	0.10, 0.17	0.14	0.10, 0.20	0.14	0.10, 0.20

		*P* < 0.001		*P* < 0.001		*P* = 0.001		*P* = 0.499		*P* < 0.001		*P* < 0.001
Skin type												
I	1.00		1.00		1.00		1.00		1.00		1.00	1.00
II	1.34	1.18, 1.53	1.30	1.14, 1.48	1.13	0.99, 1.28	1.03	0.90, 1.17	1.95	1.60, 2.37	1.88	1.54, 2.30
III	1.50	1.27, 1.76	1.41	1.18, 1.67	1.39	1.18, 1.64	1.12	0.94, 1.34	2.44	1.94, 3.06	2.23	1.75, 2.84
IV	1.16	0.71, 1.88	1.14	0.69, 1.88	0.99	0.62, 1.57	0.86	0.52, 1.42	1.65	0.88, 3.13	1.64	0.85, 3.19

		*P* = 0.021		*P* = 0.029		*P* < 0.001		*P* = 0.001		*P* = 0.016		*P* = 0.092
Ethnicity												
NZ European	1.00		1.00		1.00		1.00		1.00		1.00	
M$\overset{-}{\text{a}}$ori	1.29	1.05, 1.59	1.08	0.87, 1.34	2.08	1.66, 2.61	1.54	1.22, 1.96	1.26	0.96, 1.64	0.83	0.63, 1.10
Pacific	1.17	0.81, 1.70	0.87	0.59, 1.29	2.10	1.41, 3.13	1.31	0.86, 2.00	1.32	0.84, 2.10	0.73	0.45, 1.19
Asian	0.75	0.56, 1.00	0.62	0.45, 0.84	1.36	1.02, 1.80	1.04	0.76, 1.42	1.46	1.05, 2.04	0.93	0.64, 1.33
Other	1.31	0.76, 2.27	1.15	0.65, 2.02	0.76	0.43, 2.61	0.57	0.32, 1.04	0.33	0.10, 1.08	0.25	0.08, 0.82

*Adjusted or all six ProTan scale components, that is, all those in Tables 2 and 3, inclusive.

**Table 4:** Unadjusted and adjusted* effects with 95% confidence intervals for total Protan scores.

	Unadjusted			Adjusted (*n* = 5,392)		
		*P* = 0.004			*P* = 0.142	
Year (summer)						
1994	0.00			0.00		
1997	−0.04	−0.54, 0.45		0.08	−0.40, 0.56	
1999/2000	−0.13	−0.63, 0.37		0.59	0.10, 1.07	
2002/2003	−0.49	−0.99, 0.00		0.28	−0.21, 0.76	
2005/2006	−0.80	−1.29, −0.31		0.19	−0.29, 0.68	

		*P* = 0.005			*P* = 0.006	
City (N to S)						
Auckland	0.00			0.00		
Hamilton	−0.30	−0.79, 0.19		−0.36	−0.84, 0.11	
Wellington	−0.40	−0.89, 0.09		−0.47	−0.94, 0.01	
Christchurch	−0.94	−1.43, −0.45		−0.89	−1.36, −0.42	
Dunedin	−0.37	−0.87, 0.12		−0.29	−0.77, 0.19	

		*P* < 0.001			*P* < 0.001	
Sex						
Male	0.00			0.00		
Female	−0.77	−1.08, −0.46		−0.56	−0.86, −0.26	

		*P* < 0.001			*P* < 0.001	
Age group (years)						
15–19	0.00			0.00		
20–29	−1.70	−2.23, −1.17		−1.64	−2.16, −1.11	
30–39	−3.38	−3.90, −2.86		−3.34	−3.86, −2.82	
40–49	−3.72	−4.27, −3.17		−3.69	−4.24, −3.13	
50–59	−4.30	−4.86, −3.73		−4.22	−4.80, −3.65	
60–69	−5.52	−6.17, −4.87		−5.49	−6.16, −4.83	

		*P* < 0.001			*P* < 0.001	
Skin type						
(most sun sensitive) I	0.00			0.00		
II	1.83	1.46, 2.20		1.63	1.27, 1.99	
III	2.12	1.64, 2.60		1.88	1.40, 2.36	
(least sun sensitive) IV	−0.73	−2.21, 0.75		−0.48	−1.93, 0.97	

		*P* = 0.016			*P* < 0.001	
Ethnicity						
NZ European	0.00			0.00		
M$\overset{-}{\text{a}}$ori	0.67	0.04, 1.30		−0.48	−1.09, 0.14	
Pacific	0.62	−0.48, 1.73		−1.19	−2.26, −0.11	
Asian	−0.97	−1.82, −0.12		−2.17	−3.02, −1.32	
Other	−0.92	−2.60, 0.79		−1.74	−3.35, −0.13	

*Adjusted for all other variables in the table.
